# Recent Advances in Lupus B Cell Biology: PI3K, IFNγ, and Chromatin

**DOI:** 10.3389/fimmu.2020.615673

**Published:** 2021-01-14

**Authors:** Maria A. Bacalao, Anne B. Satterthwaite

**Affiliations:** ^1^ Department of Internal Medicine, University of Texas Southwestern Medical Center, Dallas, TX, United States; ^2^ Department of Immunology, University of Texas Southwestern Medical Center, Dallas, TX, United States

**Keywords:** lupus, B cell, autoimmunity, tolerance, PI3K, IFNγ, chromatin

## Abstract

In the autoimmune disease Systemic Lupus Erythematosus (SLE), autoantibodies are formed that promote inflammation and tissue damage. There has been significant interest in understanding the B cell derangements involved in SLE pathogenesis. The past few years have been particularly fruitful in three domains: the role of PI3K signaling in loss of B cell tolerance, the role of IFNγ signaling in the development of autoimmunity, and the characterization of changes in chromatin accessibility in SLE B cells. The PI3K pathway coordinates various downstream signaling molecules involved in B cell development and activation. It is governed by the phosphatases PTEN and SHIP-1. Murine models lacking either of these phosphatases in B cells develop autoimmune disease and exhibit defects in B cell tolerance. Limited studies of human SLE B cells demonstrate reduced expression of PTEN or increased signaling events downstream of PI3K in some patients. IFNγ has long been known to be elevated in both SLE patients and mouse models of lupus. New data suggests that IFNγR expression on B cells is required to develop autoreactive germinal centers (GC) and autoantibodies in murine lupus. Furthermore, IFNγ promotes increased transcription of BCL6, IL-6 and T-bet in B cells, which also promote GC and autoantibody formation. IFNγ also induces epigenetic changes in human B cells. SLE B cells demonstrate significant epigenetic reprogramming, including enhanced chromatin accessibility at transcription factor motifs involved in B cell activation and plasma cell (PC) differentiation as well as alterations in DNA methylation and histone modifications. Histone deacetylase inhibitors limit disease development in murine lupus models, at least in part *via* their ability to prevent B cell class switching and differentiation into plasma cells. This review will discuss relevant discoveries of the past several years pertaining to these areas of SLE B cell biology.

## Introduction

Systemic Lupus Erythematosus (SLE) is a systemic autoimmune disease that can have manifestations in multiple organ systems, including skin, musculoskeletal, cardiac, pulmonary, hematologic, and renal. SLE has a prevalence of 1:1,000 and a 10:1 female: male ratio (1). It is most common in women aged 20–40 (2), predominantly in those of African and Latin American heritage (3). It is associated with a threefold risk of death (4). The production of antibodies reactive to a variety of cellular antigens, particularly those containing nucleic acids, by autoreactive B cells is key to the development of SLE. These antibodies form immune complexes which subsequently promote inflammation and tissue damage (5). Understanding mechanisms of B cell dysregulation is therefore critical for understanding the pathophysiology of SLE as a whole and may reveal new therapeutic approaches.

B cells normally undergo both central and peripheral tolerance mechanisms which eliminate and inactivate autoreactive B cells (6–10). In the bone marrow, autoreactive immature B cells undergo receptor editing, rearranging their Ig light chains again to hopefully acquire a non-autoreactive specificity. Cells that remain autoreactive after this process are deleted by apoptosis or rendered anergic and thus unable to respond to activating stimuli. Anergy and clonal deletion also play a role in peripheral B cell tolerance. More uniquely, inactivation by inhibitory receptors and the elimination of autoreactive B cells that emerge as a result of somatic hypermutation in germinal centers (GCs) are also important. Many of these tolerance checkpoints are impaired in murine lupus models and SLE patients (6–10). As described below, excessive signaling through the PI3K pathway can result in a breach of B cell tolerance.

Abnormal B cell activation *via* signaling from both the B cell antigen receptor (BCR) and Toll-like receptors (TLR) is also crucial for SLE pathogenesis. These signals function together in the initial activation of autoreactive B cells, and also help in breaching tolerance to self-antigens (11). TLRs are expressed in B cells, where they can recognize microbial invaders. In SLE however, the endosomal TLRs 7, 8, and 9 that typically would recognize microbial DNA and RNA will also recognize and be activated by self-nucleic acids. B cells reactive with antigens that contain nucleic acids thus receive signals through both the BCR and TLRs (11, 12). While TLR9 is required for the production of antibodies against DNA, it is surprisingly protective in murine lupus models (11, 12). TLR7 plays an important pathogenic role; it is required in B cells for the formation of autoantibodies and GCs in murine lupus models, and its overexpression dramatically enhances the development of autoimmunity (11, 12).

Also important in dysregulated B cell activation in lupus are altered cytokine levels (13). BAFF (also known as BlyS) is a TNF-family ligand that promotes B cell survival and is elevated in SLE patients (5, 11, 13). SLE patients also demonstrate an “interferon signature” indicative of elevated signaling by type 1 interferons (IFNs), IFNα and IFNβ (13, 14). B cell responsiveness to TLR7 is enhanced by type 1 IFNs in both mouse and human (15, 16). IFNγ is also elevated in SLE (13, 17), and as reviewed below, also plays a crucial role in B cells for the production of autoantibodies.

The above mechanisms, among others, lead to differences in peripheral blood B cell subsets between SLE patients and healthy controls. CD19+CD27- naïve B cells are decreased, while CD19+CD27+ memory cells are relatively increased, in SLE patients (18). CD27hi plasma cells are elevated in SLE patients and correlate with disease activity (19). Lastly, CD27-IgD- (double negative, or DN cells) are also increased in SLE. DN1 cells (CXCR5+CD21+) are the more prominent DN population in healthy controls, but DN2 cells (CXCR5-CD21-CD11c+) are the more prominent compartment in SLE (20). DN2 cells are an important effector B cell subpopulation for extrafollicular plasma cell (PC) differentiation and are thought to contribute to the autoantibody pool in SLE (20–22). A similar CD11c+ population, age-associated B cells (ABC), accumulate in aging mice and are prematurely expanded in mice by autoimmune disease and chronic viral responses (23, 24).

The alterations in B cell tolerance, B cell activation and B cell subsets as well as the pathogenic role of autoantibodies suggests that targeting B cells should be an effective treatment for SLE. Indeed, Belimumab, a monoclonal antibody against BAFF, was the first drug approved for SLE since 1955 (25, 26). However, two other B cell targeted therapies - B cell depletion with the anti-CD20 antibody Rituximab and enhancement of the inhibitory activity of CD22 with Epratuzumab - were initially promising (27, 28) but each failed to meet primary endpoints in two randomized controlled trials (26, 29, 30). Several other B cell directed approaches targeting CD20, the BAFF pathway, or CD19 have either not met their primary endpoint, had mixed results, or were stopped due to adverse events (29, 31, 32). This suggests that a more nuanced understanding of B cell defects in lupus is required to develop more effective therapeutic approaches.

The past few years have provided new insights into molecular events that contribute to the initial loss of B cell tolerance and the subsequent inappropriate activation of autoreactive B cells in lupus. While space limitations preclude us from reviewing all of these novel findings, we focus here on areas of progress which have recently been studied in depth from multiple perspectives. These include the role of PI3K signaling in B cell tolerance, a requirement for B cell intrinsic interferon gamma (IFNγ) signaling for autoantibody production, and altered chromatin accessibility in lupus B cells.

## PI3K Signaling in the Loss of B Cell Tolerance

PI3K signaling plays an important role in many aspects of B cell development and activation (33–37). During B cell development, tonic signaling through PI3K promotes the positive selection of B cells that have successfully assembled a functional, but non-autoreactive, BCR (38). In the periphery, the B cell activating receptors BCR, CD40, and TLRs all engage the PI3K pathway (35–37). The strength of PI3K signaling also shapes B cell responses, with stronger signals limiting class switching (39).

**Figure 1:**
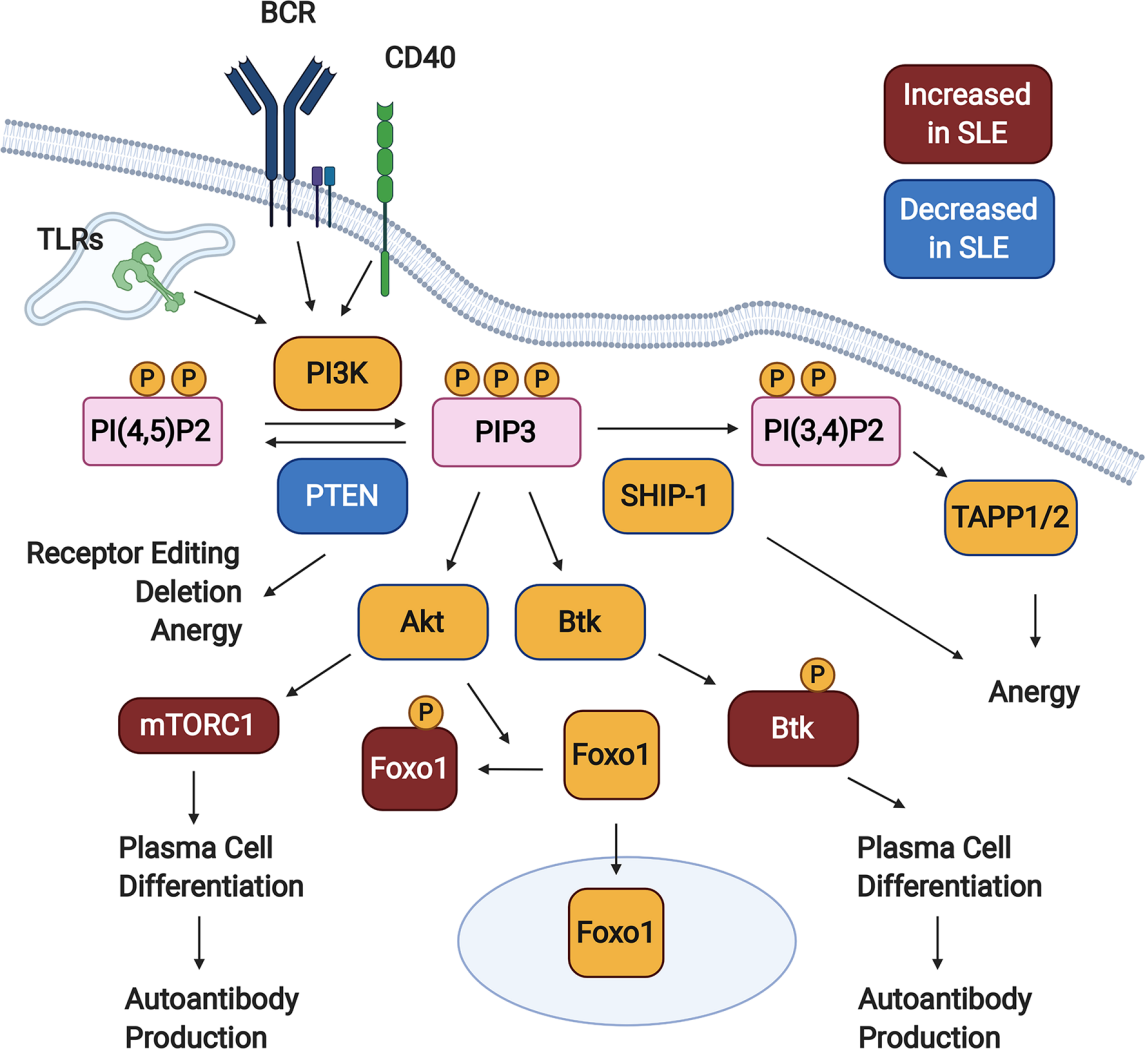
PI3K signaling promotes loss of B cell tolerance. In response to activating signals, PI(4,5)P2 is phosphorylated by PI3K, generating PIP3. Downstream of PIP3, various signaling pathways are activated (not all shown), including those mediated by Akt and Btk. PI3K signaling is kept in check by the inositol phosphatases SHIP-1 and PTEN, which dephosphorylate PIP3 to form PI(3,4)P2 and PI(4,5)P2, respectively. Studies in animal models demonstrate that PTEN promotes both central and peripheral B cell tolerance checkpoints, while SHIP-1 plays a predominant role in maintaining anergy. B cells from Systemic Lupus Erythematosus (SLE) patients demonstrate some defects indicative of elevated PI3K signaling, including reduced PTEN expression, increased cytoplasmic Foxo1, increased mTORC1 activation, and increased phosphorylation of Btk. Created with BioRender.com.

Phosphorylation of PI(4,5)P2 (PIP2) by PI3K forms the product PI(3,4,5)P3 (PIP3), creating docking sites for various signaling molecules at the plasma membrane (33, 34). This promotes their activation and facilitates access to downstream substrates and effectors. Two major pathways downstream of PI3K are mediated by the kinases Akt and Btk (**Figure 1**). It has long been known that Btk signaling promotes autoimmune disease in murine lupus models – this is reviewed extensively elsewhere (40–44). PI3K signaling is limited by two inositol phosphatases, SHIP-1 and PTEN. These dephosphorylate PIP3, with PTEN directly counteracting PI3K to form PI(4,5)P2 and SHIP-1 generating PI(3,4)P2. Over the last decade, we have learned that an appropriate balance of PI3K and SHIP-1 and/or PTEN activity is required to maintain both peripheral (anergy) and central (receptor editing and deletion) B cell tolerance (**Figure 1**). Inappropriately elevated PI3K activity in B cells promotes the production of autoantibodies in mice and is observed in B cells from SLE patients (**Figure 1**).

Studies in mouse models demonstrate an important contribution of SHIP-1 to peripheral B cell tolerance. Deletion of SHIP-1 throughout the B lineage results in a lupus-like autoimmune disease, with IgG autoantibodies focused toward nucleic acid containing antigens, Ig deposition in kidneys, and premature mortality (45). This is due primarily to the role of SHIP-1 in the maintenance of B cell anergy. Anergic B cells from two different mouse models –Ars/A1 Ig transgenic mice in which B cells recognize ssDNA, and the MD4 x ML5 model in which hen egg lysozyme (HEL) specific B cells recognize soluble HEL expressed as a self-antigen - demonstrate constitutive phosphorylation of SHIP-1 (45), indicative of increased inhibitory signaling (46, 47). Furthermore, B cell-specific SHIP-1-deficiency results in a loss of B cell anergy in both models (45, 48). Continuous SHIP-1 signaling is required to maintain tolerance, as acute deletion of SHIP-1 resulted in a rapid loss of anergy in the Ars/A1 system (49). Either induced loss of PTEN or induced expression of a constitutively active form of PI3K had the same effect, suggesting that SHIP-1 promotes anergy by limiting PI3K signaling (49). Indeed, a low dose of the PI3K inhibitor idelalisib restored anergy in Ars/A1 B cells heterozygous for both SHIP-1 and PTEN (50). In addition to limiting the levels of PIP3, SHIP-1 may also promote B cell anergy *via* its product, PI(3,4)P2. PI(3,4)P2 recruits the TAPP family of adaptor proteins to the plasma membrane. Mice expressing mutants of these adaptors that cannot bind to PI(3,4)P2 develop spontaneous germinal centers (GCs), ANAs and anti-DNA IgG, Ig deposition in the kidneys, and glomerulonephritis (51, 52). Two recent studies have suggested additional possible roles for SHIP-1 in limiting autoimmunity. When SHIP-1 is deleted in activated B cells using Aicda-cre, IL-10 expressing B cells are reduced. This may contribute to the observed autoantibody production in these animals (53). CD19-cre.SHIPf/f mice demonstrate an increase in CD11c+T-bet+ age associated B cells (ABCs), which are similar to the DN2 B cells that accumulate in SLE patients (54). These studies indicate that SHIP-1 is critical for maintaining peripheral B cell tolerance in mice.

PTEN can also promote B cell anergy in both mice and humans. Its expression is elevated in anergic B cells from healthy human subjects, and its inhibition restores normal responses to these cells (55). PTEN is also upregulated in anergic B cells in the MD4 x ML5 mouse model (45, 56), and its deficiency in B cells causes loss of tolerance in this system. Combined heterozygosity of both PTEN and SHIP-1 impairs anergy in Ars/A1 B cells (50), as does acute deletion of PTEN (49). 3-83ki mice, which carry a BCR transgene that recognizes the self-antigen MHC Class I H2-K^b^, demonstrate that PTEN is not always necessary for the induction of anergy but reveal roles for PTEN in additional B cell tolerance mechanisms. Both receptor editing and deletion are impaired when B cells either lack PTEN or express a constitutively active PI3K alpha catalytic subunit in the 3-83ki system (57, 58). Autoreactive cells that escape receptor editing and deletion accumulate as anergic cells (57). Consistent with these observations, PTEN-deficient immature B cells are resistant to apoptosis induced by BCR crosslinking (59). Furthermore, loss of PTEN prevents B cell deletion in an IgM^b^ macroself model, in which mice express a superantigen that binds to IgM and induces apoptosis of immature B cells *in vivo* (60, 61). Overexpression of either of the miRNAs miR-19 or miR-148a, which target PTEN, also breaches tolerance in the IgM^b^ macroself model (60, 61). Thus, PTEN contributes to multiple mechanisms of B cell tolerance.

Taken together, these findings suggest that lupus B cells may demonstrate inappropriate activation of PI3K signaling. Indeed, increased phosphorylation of Akt, which occurs downstream of PI3K, is observed in B cells from Sle1.Sle3, BXSB, MRL.lpr, and Lyn-/- mice, all of which develop lupus (62, 63). Several recent observations suggest that PI3K signaling is also elevated in B cells from at least a subset of SLE patients. A study of treatment naïve SLE patients compared to healthy controls showed reduced levels of PTEN expression in most B cell subsets, except for memory cells (64). PTEN levels were inversely correlated with disease activity (64). Reduced PTEN levels in SLE B cells were due at least in part to increased expression of miRNAs, including miR-7, miR-21, and miR-22, that limit PTEN expression (64). Downstream of PI3K, the transcription factor Foxo1 is phosphorylated by Akt which results in its exclusion from the nucleus. Consistent with increased PI3K activity, cytoplasmic Foxo1 was shown to be elevated in SLE B cells (65). This is particularly pronounced in IgD- CD27- DN B cells and correlates with disease activity (65). Another function of PI3K signaling is activation of the mTORC1 pathway. This pathway was shown to be elevated in a CD11b+T-bet+ “atypical memory” population in SLE patients similar to DN2 cells that accumulate in correlation with disease activity and anti-dsDNA antibodies (66). This has functional consequences, as the mTORC1 inhibitor rapamycin prevents both the development of these cells *in vitro* and their differentiation into plasma cells (PCs) (66). Rapamycin also prevents the development of newly generated PCs and reduces autoantibody production in the NZB/W murine lupus model (67). Finally, phosphorylation of Btk, which is downstream of PI3K and promotes autoimmunity in mice (40–44), was found to be increased in SLE B cells relative to healthy controls (68).

The correlation between heightened PI3K signaling in SLE patients’ B cells and disease activity suggests that this may be a consequence, rather than a cause, of increased B cell activation. The recent characterization of patients with activating mutations in the PI3K delta catalytic subunit allows an understanding of causal roles for PI3K signaling in human autoimmunity. These patients have a combined immunodeficiency which is associated with some form of autoimmunity in 28% of cases (69). Both IgM autoantibodies and B cells expressing VH4-34, which confers autoreactivity, are increased in these patients (70). Thus, hyperactive PI3K signaling can contribute to a loss of immune tolerance in humans. However, only a fraction of these patients with autoimmune involvement have SLE like or other rheumatic disease like symptoms (69), suggesting a general rather than disease specific role for PI3K in promoting human autoimmunity. Mice carrying an activating mutation of PI3K delta seen in these patients demonstrate a loss of B cell tolerance in the HEL system. The induction of anergy is impaired and mice accumulate autoreactive marginal zone B cells, PCs and GC B cells, although high affinity autoreactive GC B cells are selected against (70). An independently generated mouse strain with the same mutation developed autoimmunity in a manner dependent on the microbiota (71), suggesting that PI3K signaling may provide a link between gene/environment interactions in the development or amplification of autoimmune disease.

## IFNγ Signaling in B Cells Promotes Autoimmunity

Much attention has been paid to the role of type 1 interferons in the pathogenesis of SLE (13, 14). However, of late there has been a resurgence in the study of interferon gamma (IFNγ) in lupus, particularly in the context of B cell responses. IFNγ has long been known to promote autoimmunity and nephritis in several murine lupus models, including NZB x NZW F1 mice, MRL.lpr mice, and pristane treated mice (17, 72). IFNγ is also elevated in the serum of SLE patients (17). Recent studies using patient serum samples collected prior to the diagnosis of SLE have shown that this increase in IFNγ occurs coincident with the appearance of autoantibodies and prior to the development of clinical symptoms (73). ANA+ healthy individuals also demonstrate increased IFNγ levels, although lower than those in SLE patients (74). This suggests that IFNγ may be involved in the initial loss of B cell tolerance early in the development of lupus.

Two genetically distinct mouse lupus models have recently been used to demonstrate a B cell intrinsic requirement for IFNγ receptor (IFNγR) expression in order to develop autoreactive GCs, produce autoantibodies, and undergo kidney damage. The first is a bone marrow chimera model in which B cell deficiency of Wiskott-Aldrich syndrome protein leads to lupus-like autoimmunity (75). The second involves the Sle1b lupus susceptibility allele, either alone (76), which breaches B cell tolerance, or in the context of enhanced TLR7 signaling (77), which leads to full blown autoimmune disease. Of potentially significant therapeutic importance, IFNγ was not required for B cells to respond to immunization with either model antigens (76) or virus like particles (VLPs) (75). Phosphorylation of Stat1 at S727 is similarly required in B cells for autoimmunity in the Sle1b + TLR7 model, but not for responses to foreign antigens (78). Stat1 S727 can be phosphorylated downstream of both IFNγ and TLR7, suggesting that these signals may converge on Stat1 to promote autoantibody production (78).

**Figure 2:**
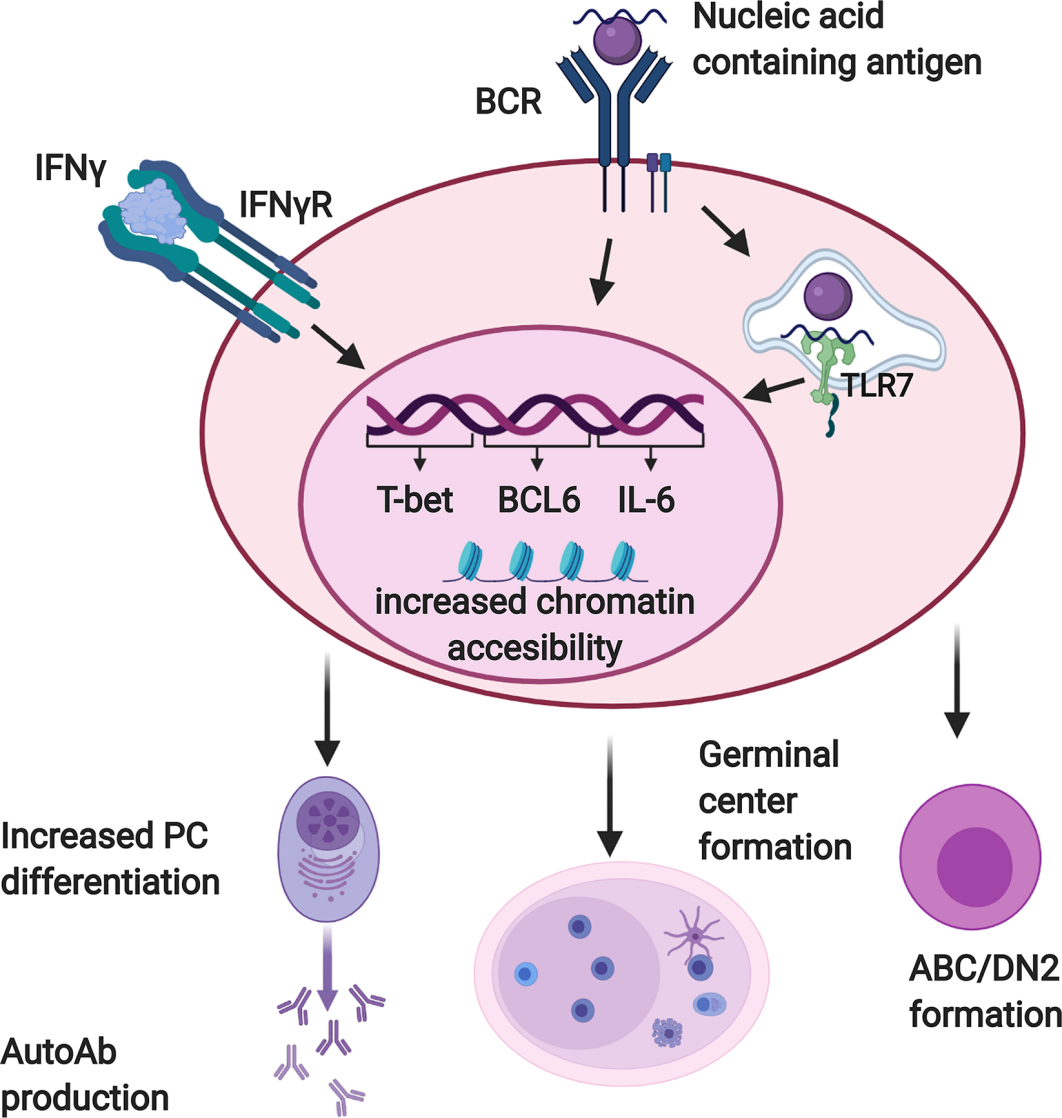
Interferon γ (IFNγ) promotes autoreactive B cell activation and differentiation. B cells reactive with nucleic acid containing antigens receive dual activating signals *via* the BCR and endosomal, nucleic acid sensing Toll-like receptors (TLRs) including TLR7. Autoreactive B cells must also respond to IFNγ in order to produce autoantibodies and form germinal centers. When these three signals are received together, B cells upregulate factors which promote autoantibody production and germinal center formation, including T-bet, BCL-6, and IL-6. They also demonstrate increased chromatin accessibility at motifs for transcription factors that promote differentiation of DN2/ABC cells and plasma cells. Created with BioRender.com.

IFNγ has numerous effects on B cells that contribute to the production of autoantibodies (**Figure 2**). It enhances the expression of IL-6 (79) and the transcription factors Bcl-6 (75) and T-bet (75, 76, 80, 81) by B cells responding to stimulation through the BCR in conjunction with TLR7, with or without CD40 engagement. Bcl-6 is critical for GC formation (75), and B cell-derived IL-6 is required for autoimmunity in the WAS chimera model (79). T-bet contributes to GCs and autoantibody production in some (76, 82, 83), although not all (75), lupus models. It also promotes PC differentiation of B cells activated in the presence of IFNγ (84). T-bet is also expressed in ABCs and DN2 cells, populations of CD11c+ B cells that accumulate in murine lupus models and human SLE patients, respectively, and differentiate efficiently into autoantibody secreting cells (20–24). IFNγ promotes both the development and terminal differentiation of DN2 cells *in vitro* (20, 82). Stimulation of human naïve B cells with anti-IgM, R848 (a TLR7/8 ligand), and IFNγ induces a T-bet+ DN (IgD-CD27-) population in an IFNγ-dependent manner (82). IFNγ stimulation in this context sensitizes human B cells to R848 and also primes cells for responsiveness to IL-21 by increasing IL-21R expression (82). IL-21 and R848 subsequently promote differentiation of the T-bet+ DN cells into antibody secreting PCs (22, 82). *In vivo*, systemic IFNγ levels and T-bet+ DN2 cells are correlated in SLE patients (82). Taken together, these observations suggest that IFNγ signaling in B cells contributes to autoimmunity by promoting the development of spontaneous GCs as well as supporting the development and subsequent differentiation of ABCs and DN2 cells.

IFNγ likely exerts at least some of these effects by modulating B cell chromatin accessibility. In the context of stimulation through the BCR and TLR7, IFNγ and IL2 act synergistically to expand chromatin accessibility in human B cells *in vitro* (82). Accessibility was enhanced around T-bet, STAT5, NFkB, IRF4 and Blimp1 motifs, transcription factors needed for B cell differentiation into PCs. Chromatin accessibility was also enhanced around the IL-21 receptor locus. Stone et al. further evaluated the role that IFNγ-induced transcription factors had on PC development (84). Mouse B cells activated *in vitro* in the presence of Th1 cells, which secrete IFNγ, differentiated more efficiently into PCs than did those activated in the presence of Th2 cells, which do not express IFNγ. This depended on T-bet and B cell-intrinsic expression of the IFNγR, and was associated with increased chromatin accessibility at T-bet, IRF1, and Blimp-1 motifs. IFNγR signaling in B cells increased expression of both Blimp1 and T-bet, which promote PC differentiation in this context *via* distinct mechanisms. Blimp1 does so directly, while T-bet acts indirectly by limiting the transcription of pro-inflammatory genes that would otherwise reduce PC differentiation if unchecked. These studies suggest that IFNγ promotes changes in chromatin accessibility that prime B cells for subsequent differentiation into PCs, and may explain why ABCs and DN2 cells differentiate so efficiently.

Several factors have been found to facilitate the ability of B cells to respond to IFNγ in lupus. TLR7 stimulation increases B cell expression of IFNγR (77). Furthermore, IFNγ production by CD4+ T cells is enhanced by several lupus associated stimuli. B cell intrinsic defects that induce autoimmunity such as WAS-deficiency (75), constitutive activation of Btk (85), or galectin-3 deficiency (86) can drive IFNγ production by T cells in a manner dependent on B/T interaction. Topical treatment with the TLR7 ligand imiquimod leads to increased IFNγ expression by Tfh cells *in vivo* (77). BAFF can also act directly on T cells to increase IFNγ expression in the Lyn-/- model of lupus (87). Thus, in the lupus prone environment B cells can have both increased access and responsiveness to IFNγ.

Given the known association between type I IFNs and lupus, several recent studies compared the effects of IFNα and IFNγ. In both the WAS chimera (75) and the Sle1b + imiquimod mouse models (77), IFNα deficiency reduced, but did not completely abrogate, the development of autoimmunity. In contrast, IFNγ was absolutely required (75, 77). In humans, IFNγ was elevated earlier in the development of SLE than IFNα (73). The former preceded or was coincident with the appearance of autoantibodies, while the latter occurred after the acquisition of autoantibodies but prior to disease diagnosis (73). In ANA+ healthy individuals, IFNγ, but not IFNα, is increased relative to ANA- healthy controls (74). While autoimmune side effects have not been a major complication of therapeutic use of recombinant IFNγ (88, 89), there are reports of this treatment resulting in increased autoantibodies (90–94), suggestive of a role for IFNγ in promoting a loss of B cell tolerance in susceptible individuals. Taken together, these observations suggest a scenario in which B cell exposure to IFNγ contributes to the initial loss of B cell tolerance, while IFNα serves to amplify the subsequent inflammation that drives clinical disease.

## Altered Chromatin Accessibility in SLE B Cells

IFNγ drives both dramatic changes in chromatin accessibility and the production of pathogenic autoantibodies, highlighting the potential importance of epigenetic control of autoimmunity. Several recent studies have revealed alterations in chromatin accessibility in B cells from SLE patients and elucidated mechanisms by which histone modifiers affect B cell responses in autoimmune disease.

Recent studies of chromatin accessibility in SLE B cells have focused primarily on two measures: DNA methylation and ATAC-Seq. DNA methylation between paired CG groups leads to DNA compaction and decreased transcription (95). Perturbation in methylation increases apoptosis, leading to a release of apoptotic DNA that can trigger autoimmunity. DNA hypomethylation has been noted to trigger lupus-like conditions in mice. SLE patients have been noted to have altered DNA methylation as well (95). Hypomethylation is observed in the vicinity of type 1 IFN-regulated genes in lupus, consistent with the characteristic IFN signature (96–99). A recent study (100) found that ethnicity influences these DNA methylation patterns. They were most pronounced in African American SLE patients compared to healthy women and were apparent as early as the transitional B cell stage in African American patients. A large number of other genes demonstrate increased methylation in all subpopulations of SLE B cells tested compared to healthy controls (96, 98). Thus, complex global changes in DNA methylation are observed in lupus B cells and may contribute to aberrant B cell responses.

Scharer et al. have recently used ATAC-seq to elucidate changes in chromatin accessibility in SLE B cell subsets (96, 101). In naïve B cells from healthy volunteers, chromatin accessibility was enhanced at NRF1, CTCF and STAT5 binding sites (101). In naïve B cells from the SLE cohort, chromatin accessibility was instead enhanced at motifs for transcription factors involved in B cell activation and differentiation, namely NFκB, AP-1, BATF, IRF4, and PRDM1 (101). The epigenetic changes in resting naïve B cells in SLE were also present in other B cell subsets (96). As resting naïve B cells represent the earliest B cells available for an immune response, this data suggests that epigenetic dysregulation in SLE may occur early in B cell development. This study also demonstrated that DN2 cells have enhanced chromatin accessibility in genes involved in BCR and TLR signaling as well as costimulatory molecules in both healthy and SLE volunteers (96). DN2 cells were found to have greater accessibility at T-bet, EGR, and AP-1 motifs in comparison to other B cell subsets, while NFκB sites are less accessible in DN2 cells compared to naïve B cells (96). Similarly, the accessible chromatin structure in ABCs from lupus prone mice is enriched in AP-1, IRF and T-bet motifs (102). The increased accessibility at T-bet binding sites in DN2 cells and ABCs is consistent with a role for IFNγ in the development of these subpopulations. EGR family members, AP-1 family members, and the AP-1 superfamily member Atf3 are upregulated in SLE B cells. EGR family members and Atf3 are predicted to regulate many of the genes that are differentially expressed between SLE patients and healthy control B cells (96). These observations suggest that AP-1 and EGR work in conjunction with T-bet to shape the unique epigenome of SLE B cells.

Consistent with altered chromatin structure in lupus B cells, histone modifiers affect the development of autoimmune disease in murine lupus models. The modification of histones on amino acid tails is one of the most important mechanisms that influence chromatin structure and accessibility. A variety of post-translational modifications on histones can regulate gene transcription, both positively and negatively. Key among these is histone acetylation. Acetylation of lysine residues within histones relaxes chromatin structure by neutralizing its positive charge, increasing the accessibility of the regulated gene for transcription factors (103). Acetylation is governed by various histone acetyltransferase (HAT) enzymes. Consequently, histone deacetylation, mediated by various histone deacetylase (HDAC) enzymes, leads to chromatin compaction, reduced chromatin accessibility and decreased gene transcription. Early studies demonstrated that HDAC inhibitors suberoylanilide hydroxamic acid and Trochostain A decrease renal disease and inflammatory cytokine production in the MRL/*lpr* and NZB/NZW F1 murine lupus models (104). More recent reports have revealed pathogenic roles for HDAC6 and HDAC9 in NZB/W and MRL.lpr mice, respectively (105–109). Here we review new advances in our understanding of how histone modifiers alter B cell responses in autoimmune disease.

Several recent reports define a role for HDACs in promoting B cell differentiation and class switching. Treating MRL/*lpr* mice with the HDAC inhibitor parabinostat reduced autoreactive PC and autoantibody production, leading to a reduction in nephritis (110). Parabinostat reduced the proliferation, survival, and PC differentiation of purified murine B cells *in vitro* in response to both T-independent and T-dependent stimuli (110). The short chain fatty acids (SFCAs) valproic acid (VPA), butyrate and proprionate act as classical HDAC inhibitors. They have potential for clinical use as VPA is an FDA approved drug and butyrate and proprionate are derived from the processing of dietary fiber by the microbiota. Treatment of MRL.lpr mice with either VPA or butyrate plus proprionate reduced autoantibodies, prevented kidney damage and skin lesions, and increased survival (111, 112). Importantly, VPA was effective in both prevention and treatment studies (112). SFCA were shown to decrease class switch recombination (CSR), somatic hypermutation (SHM) and PC differentiation in mice *in vivo*, and CSR and PC differentiation of both murine and human B cells *in vitro* (111, 112). This effect was more specific than that of parabinostat (110), as it was observed under conditions that did not impair B cell proliferation or survival (112). SFCAs reduce expression of both Blimp-1 (encoded by Prdm1), which is required for PC differentiation, and activation-induced cytidine deaminase (AID, encoded by Aicda), which is necessary for CSR and SHM (111, 112). SFCAs do not target these genes directly, but rather promote the upregulation of several miRNAs that subsequently downregulate Prdm1 (miR-23b, miR-30a, and miR-125b) and Aicda (miR-155, mir-181b, and miR-361) (112). SCFAs were shown to exert their effect on B cells by inhibiting HDACs rather than by acting as energy substrates or acting through G-protein coupled receptors (111).

Estrogen may also play a role in the epigenetic dysregulations occurring in SLE, which may partially explain the strong female predominance of the disease. Estrogen boosts the production of mature antibodies by promoting the expression of AID (113). A recent study by Casali et al. demonstrated that estrogen counteracts the activity of HDAC inhibitors on the class switching of mouse B cells and the production of autoantibodies (114). Mechanistically, estrogen reversed the HDAC inhibitor-mediated upregulation of miR26-A, which targets Aicda, the gene that encodes AID (114).

Inhibition of classical HDACs thus leads to amelioration of autoimmune disease in lupus models, at least in part due to a reduction of PC differentiation and class switching by B cells. These HDACs thus play a pathogenic role in lupus. Intriguingly, the non-classical HDAC Sirtuin1 (Sirt1) was recently demonstrated to have the opposite effect (115). Sirt1 was expressed in resting murine and human B cells. Its levels were decreased in response to stimuli that induce AID expression *in vitro* and in B cells that had elevated AID expression in both female MRL/Fas^lpr/lpr^ mice and SLE patients. Deletion of Sirt1 specifically in activated murine B cells led to an increase in class-switched autoantibodies against dsDNA, histones, ribonucleoprotein (RNP), and RNA. In contrast, activation of Sirt1 reduced autoantibody levels in MRL.lpr mice. *In vitro*, Sirt1-deficient B cells demonstrated enhanced class switching and increased AID expression, while PC differentiation and Blimp-1 expression were unaffected. Sirt1 was shown to deacetylate histones, Dnmt1, and NFκB at the Aicda promoter. Thus, AID expression is tightly controlled by complex epigenetic mechanisms, an appropriate balance of which is required to limit autoantibody production.

In addition to affecting B cell differentiation and class switching, epigenetic modifiers have been shown to control B/T cognate interactions during autoimmunity. Tet2 and Tet3 can both demethylate DNA and recruit HDACs (116). B cell specific deletion of both Tet2 and Tet3 results in increased activation of B and T cells, autoantibody production, and a mild autoimmune disease (117). This depends on B/T interactions and results from enhanced expression of the costimulatory molecule CD86 on B cells. Tet2 and Tet3 are required for the binding of HDAC1 and HDAC2 to a CD86 enhancer. HDAC inhibitors result in increased CD86 expression on anergic cells, similar to the effect of Tet2/Tet3 deficiency.

Recent reports have also implicated enzymes involved in histone methylation in the development of lupus as well as the control of B cell activation. Histone methylation can either promote or repress transcription, depending on the position methylated. More specifically, the histone lysine methylations H3K4, H3K36, and H3K79 promote transcription while H3K9, H3K27, and H4K40 have a repressive effect (118). EZH2, a histone methyltransferase that produces H3K27me3, was found to be upregulated in CD4+ T cells, B cells, monocytes, and neutrophils in SLE patients (119, 120). Inhibiting EZH2 in MRL/lpr mice improved survival and decreased anti-dsDNA antibodies, inflammatory cytokine and chemokine levels, renal disease, and lymphoproliferation (120). The pathogenic role of EZH2 in lupus may be in part due to its promotion of PC differentiation and antibody secretion. EZH2-deficient mice demonstrate a B cell intrinsic defect in PC formation *in vivo* in response to LPS (121). EZH2-deficient B cells failed to downregulate genes involved in inflammation and the B cell (as opposed to PC) fate. Furthermore, metabolic genes required for PC fitness and antibody secretion were not upregulated in the absence of EZH2. EZH2 also promotes PC differentiation in activated human B cells. In these cells it has been shown to bind to the promoter of Bach2, a repressor of PC differentiation, and inhibit its expression *via* methylation (122). In contrast, chemical inhibition of EZH2 promoted murine PC differentiation *in vitro*, although antibody secretion was not enhanced (123). This suggests possible context dependent roles for EZH2 in these processes.

The histone demethylases KDM4A and KDM4C, which promote demethylation of H3K9 (124), limit B cell activation and proliferation in response to stimulation with Tfh-derived signals. They are upregulated upon activation and subsequently promote the expression of *Wdr5*, which then upregulates the cell cycle inhibitors *Cdkn2c* and *Cdkn3.* The levels of KDM4A and KDM4C mRNA were upregulated in B cells from healthy human donors upon activation by IL21, BAFF, and anti IgM. However, these levels were reduced in both unstimulated and stimulated B cells from SLE patients compared to the healthy controls. The mRNA levels of *WDR5* and various cell cycle inhibitors were likewise reduced in SLE B cells, suggesting that impaired upregulation of KDM4A and KDM4C may result in enhanced proliferation of SLE B cells upon activation.

## Conclusion

Recent studies have highlighted new pathways important for driving autoreactive B cell responses in lupus. These observations, which are discussed in this review and summarized in **Table 1**, highlight new mechanisms of B cell dysregulation and suggest potential new therapeutic approaches. Recent insights into PI3K signaling have elucidated its role in B cell tolerance and suggest a possible contribution to SLE pathogenesis. Studies in BCR transgenic mouse models have shown that either loss of negative regulators of the PI3K pathway or constitutive activation of PI3K can result in impaired central and peripheral B cell tolerance checkpoints. PI3K signaling is increased in SLE B cells. Aberrant PI3K signaling is correlated with disease activity. However, the degree to which PI3K signaling plays a causative role in loss of tolerance in humans is unclear, and not all humans with PI3K derangements have autoimmune disease. Subsequently, there are still unanswered questions regarding the role PI3K signaling plays in early autoimmunity and the influence it may have in tolerance checkpoints or inappropriate B cell activation in SLE patients. The therapeutic potential of the PI3K pathway also remains to be explored. Treatment of Ars/A1 mice heterozygous for both SHIP-1 and PTEN with idelalisib restored anergy in their B cells (50). Given that the PI3K pathway causes activation of the mTORC1 pathway, the mTORC1 inhibitor rapamycin may have therapeutic benefit. It has been shown to prevent the development of newly generated PCs and reduce autoantibody production in the NZB/W murine lupus model (67). Indeed, a recent open label phase 1/2 trial of sirolimus (rapamycin) demonstrated a reduction in disease activity in SLE, although B cell responses were not measured in this study (125). Another downstream component of PI3K signaling pathways in B cells, Btk, promotes the development of lupus in mouse models (40–44) and is being targeted in several clinical trials in SLE (www.clinicaltrials.gov).

**Table 1:** Differences between healthy and lupus B cells discussed in this review.

1.1.1 Domain	1.1.2 Healthy B Cells	1.1.3 Lupus B Cells
1.1.4 B cell subsets	Human: Increased CD19+CD27- naïve B cells, decreased CD19+CD27+ memory cells, decreased CD27hi plasma cells, and decreased CD27-IgD-CD11c+T-bet+ DN2 cells compared to SLE B cells Mouse: Reduced germinal center B cells, plasma cells, and ABCs (murine equivalent of DN2 cells) compared to lupus B cells	Human: Decreased CD19+CD27- naïve B cells, increased CD19+CD27+ memory cells, increased CD27hi plasma cells, and increased CD27-IgD-CD11c+T-bet+ DN2 cells compared to healthy control B cells Mouse: Increased germinal center B cells, plasma cells, and ABCs (murine equivalent of DN2 cells) compared to wild type B cells

1.1.5 PI3K signaling	Appropriate balance of PI3K and SHIP-1 and/or PTEN PTEN and SHIP promote B cell tolerance in mice PTEN is elevated in healthy human anergic B cells and its inhibition impairs anergy	Elevated PI3K signaling or deletion of SHIP-1 or PTEN promote autoimmune disease in mice PTEN is reduced and signaling events downstream of PI3K (pBtk, cytoplasmic Foxo1, mTORC1 activation) are enhanced in B cells from SLE patients compared to healthy controls

1.1.6 IFNγ	Elevated in ANA+ healthy individuals, but less so than in SLE patients Not required to signal in B cells for response to model foreign antigens	Elevated in SLE patients prior to the development of clinical disease Must signal in B cells for autoimmunity in lupus mouse models. Induces expression of T-bet and promotes ABC, DN2, and PC differentiation in conjunction with TLR7 signaling Enhances chromatin accessibility in both human and mouse B cells

1.1.7 Epigenetic Changes	Increased methylation around type 1 IFN-regulated genes In healthy naïve B cells, chromatin accessibility is enhanced at NRF1, CTCF, and STAT5 binding sites	Hypomethylation around type 1 IFN-regulated genes In naïve SLE B cells, chromatin accessibility is enhanced around NFkB, AP-1, BATF, IRF4, and PRDM1 binding sites, promoting B cell activation and differentiation. DN2 cells have increased chromatin accessibility at T-bet, EGR, and AP-1 motifs. ABCs have increased chromatin accessibility at T-bet, IRF, and AP-1 motifs. HDACs generally promote B cell class switching, PC differentiation, and autoimmunity in mouse lupus models.

Although type I interferons have long been implicated in SLE pathogenesis and have recently been successfully explored as therapeutic targets (126), IFNγ signaling has recently garnered renewed interest. IFNγ signaling to B cells is required for autoantibody production in mice and promotes the development of ABCs and DN2 cells *in vitro*. Tbet+ DN2 cells and IFNγ levels are correlated in SLE patients. IFNγ has also been implicated in early autoimmunity as its levels are elevated concurrently with autoantibody production in ANA+ healthy individuals. These observations suggest that IFNγ could prove a useful target for development of disease modifying therapeutics. However, a phase 1b clinical trial of an anti-IFNγ antibody did not demonstrate a clinical effect and did not reduce anti-dsDNA antibodies in SLE (127). This could reflect a difference in the relative importance of type 1 IFNs and IFNγ in mice vs. humans. It may also be that only subsets of SLE patients have IFNγ driven disease. Alternatively, IFNγ may play its unique and critical role during the initial loss of tolerance in the preclinical phase of SLE. If so, targeting it when patients have come to clinical attention might no longer be effective since type I IFN mediated amplification of inflammation would be dominant at that point. Therefore, strategies such as blocking IFNγ in particular subsets of patients (82), in combination with other targets (77), or at a different stage in the disease course may be more efficacious.

Lastly, chromatin accessibility has also been a fruitful area of research over the past several years. Alterations in chromatin structure have been noted in SLE B cells, with increased accessibility at motifs for transcription factors that promote B cell differentiation, autoantibody production, and the development of DN2/ABC cells. These factors include T-bet, IRF4, and Blimp1/PRDM1, among others. IFNγ signaling is one potential contributing factor. HDAC inhibitors and other modulators of chromatin accessibility block PC differentiation and CSR and subsequently reduce autoantibody production and disease activity in murine lupus models. The clinical applications of these observations are still unfolding. Some HDAC inhibitors, notably parabinostat which has powerful inhibitory effects on PC differentiation *in vitro* as well as in mouse models, may have a similar effect on human autoreactive B cells (110). Dietary fiber derived SCFAs with HDAC inhibitory activity likewise may have potential for clinical use in SLE, but their effectiveness is influenced by changes in gut microbiota (111). Further studies identifying the signals that drive lupus-associated changes in chromatin accessibility *in vivo* and defining those epigenetic changes that correlate with a robust response to treatment may illuminate more targeted therapeutic approaches for SLE.

## Author Contributions

MB wrote portions of the manuscript and designed the figures. AS wrote portions of the manuscript, modified the figures, and approved the final manuscript. All authors contributed to the article and approved the submitted version.

## Funding

This work was supported by NIH grants AI122720 and AI137746 to AS.

## Conflict of Interest

MB is a subinvestigator on clinical trials sponsored by Hoffman LaRoche, Eli Lilly, and UCB. AS holds stock in Amgen, is a Southwestern Medical Foundation Scholar in Biomedical Research and holds the Peggy Chavellier Professorship in Arthritis Research and Treatment.
